# The Relationship Between Filial Piety and the Academic Achievement and Subjective Wellbeing of Chinese Early Adolescents: The Moderated Mediation Effect of Educational Expectations

**DOI:** 10.3389/fpsyg.2022.747296

**Published:** 2022-03-17

**Authors:** Xiaolin Guo, Junjie Li, Yingnan Niu, Liang Luo

**Affiliations:** Collaborative Innovation Center of Assessment Toward Basic Education Quality, Beijing Normal University, Beijing, China

**Keywords:** filial piety, educational expectations, academic achievement, subjective wellbeing, moderated mediation effect, Chinese adolescents

## Abstract

A successful student has been defined as one who not only performs well in academics but is also happy. Hence, how to promote adolescents’ academic success and wellbeing is an important issue with which researchers have been concerned. A few studies have explored the relationship of filial piety to the academic achievement or life satisfaction of Chinese adolescents. However, in view of the close relationship between the two outcomes, the unique effects of filial piety on academic achievement and subjective wellbeing and their underlying mechanisms need to be further clarified. Based on a sample of 677 students in Grade 6 (M_age_ = 12.24, SD = 0.36) and their parents in Beijing, China, this study examines how adolescents’ reciprocal filial piety (RFP) and authoritarian filial piety (AFP) are related to their academic achievement and subjective wellbeing. It also examines the mediating role of adolescents’ educational expectations in these relationships, and the moderating role of parents’ educational expectations in the relationships of adolescents’ filial piety to educational expectations and of adolescents’ educational expectations to academic achievement and subjective wellbeing. The results indicate that, when the two outcome factors are considered simultaneously, RFP is positively related to academic achievement and subjective wellbeing. In contrast, AFP is negatively related to academic achievement but not significantly related to subjective wellbeing. Moreover, adolescents’ educational expectations play a mediating role in the relationships of both RFP and AFP to academic achievement and subjective wellbeing. In addition, the positive effect of adolescents’ educational expectations on subjective wellbeing is stronger when mothers’ educational expectations are higher, supporting the moderating role of parents’ educational expectations. Our findings provide new insights into and implications for the moderated mediation mechanism underlying the links between filial piety and early adolescent development.

## Introduction

Filial piety is a central concept in Confucianism that prescribes how children ought to behave and treat their parents and ancestors (Yeh and Bedford, 2003). It has long provided the moral basis for parent–child relationships and socialization patterns in China (Ho, 1987, 1994). Given that filial piety at the individual level reflects one’s psychological needs and interaction patterns with parents, it has been considered to have critical implications for individual development (Yeh and Bedford, 2003; Bedford and Yeh, 2019). During adolescence, academic success and happiness are regarded as two key indicators for defining a successful adolescent student (OECD, 2017). Hence, it is important to examine the relationship and mechanism between adolescents’ filial piety and their academic achievement and subjective wellbeing. This study aims to investigate the relationships of adolescents’ filial piety to academic achievement and subjective wellbeing and to test the mediating role of adolescents’ educational expectations and the moderating role of parents’ educational expectations in these relationships among a sample of Chinese early adolescents.

### Filial Piety, Academic Achievement, and Subjective Wellbeing

Filial piety is currently defined as a contextualized personality construct that is represented by a pair of culturally sensitive psychological schemas for interaction with parents (Bedford and Yeh, 2019, 2021). Reciprocal filial piety (RFP) is based on genuine affection arising from long-term equal and intimate parent–child interactions. Children with RFP tend to respect and support their parents spiritually and to take care of their parents as they age. Such filial belief is due to children’s gratitude for their parents’ investment and sacrifice. In contrast, authoritarian filial piety (AFP) advocates obedience to authority and role obligations. Children with AFP tend to comply with their parents’ wishes despite disagreeing with them, and they aim to honor the family name. Such filial belief is motivated by the desire for collective identification (Yeh, 2003, 2006; Bedford and Yeh, 2019, 2021). The two aspects of filial piety coexist within a person and function simultaneously on the person. Previous research has shown that RFP and AFP can sometimes promote the same outcome, while at other times, they might result in different outcomes (Yeh, 2003; Yeh and Bedford, 2003; Bedford and Yeh, 2019).

The implications of filial piety for Chinese adolescents’ academic success have been a concern of some researchers (Chow and Chu, 2007; Hui et al., 2011; Chen and Wong, 2014; Chen, 2016; Zhou et al., 2020). Chinese culture advocates that individual development and performance are intended to achieve not only personal but also familial success (Huang and Gove, 2015). Family obligation is highly related to Chinese adolescents’ academic motivation (Hui et al., 2011). Academic achievement is seen by Chinese students as a primary way of honoring their families and repaying their parents for their efforts and sacrifices (Ho, 1986; Chow and Chu, 2007; Tao and Hong, 2014; Fwu et al., 2016). From this perspective, the gratitude for parents’ sacrifice embedded in RFP and the sense of duty to maintain the family reputation embedded in AFP should both motivate students to strive for academic excellence and achieve better academic performance. However, because AFP emphasizes self-suppression and obedience to parental demands (Bedford and Yeh, 2019), students with AFP tend to have performance-based goal orientations rather than a focus on acquiring new knowledge and skills, and their academic achievement is not motived by their own learning beliefs (Chen and Ho, 2012; Chen, 2016). Thus, AFP may lead students to suffer from a lack of intrinsic motivation to engage in learning activities and thereby to fall behand in their academic achievements. Based on data from junior high school students in Eastern China and university students in Hong Kong (China), researchers have found that RFP was positively related to academic achievement; while AFP was negatively related to academic achievement (Chen and Wong, 2014; Chen, 2016; Zhou et al., 2020), although the strength of the relationships varied among the studies.

Furthermore, as filial piety implies underlying mechanisms in parent–child relationships (Bedford and Yeh, 2019, 2021), it may relate to adolescent wellbeing. Since RFP and AFP reflect a horizontal and vertical relationship, respectively, between parents and children, RFP was initially thought to have an overall beneficial effect and AFP have an overall harmful effect on individual wellbeing (Yeh, 2003). However, several studies found that AFP could also have positive implications for psychological adjustment, such as reducing parent–child conflict and enhancing intergenerational support (Yeh and Bedford, 2004; Yeh et al., 2013). AFP could fulfill individual psychological needs for social belonging and collective identity, although it cannot fulfill the need for relatedness (Bedford and Yeh, 2019). A few studies have consistently demonstrated a positive relationship between RFP and life satisfaction among Chinese adolescents (Leung et al., 2010; Chen et al., 2016; Yan and Chen, 2018; Jen et al., 2019; Sun et al., 2019; Leung and Shek, 2020; Wu and Chen, 2020). However, inconsistent results have been reported on the relationship between Chinese adolescents’ AFP and life satisfaction, with some studies demonstrating a positive effect (Leung et al., 2010; Yan and Chen, 2018; Leung and Shek, 2020; Wu and Chen, 2020) and others demonstrating a negative or no effect (Jen et al., 2019; Sun et al., 2019).

### Mediating Effect of Adolescents’ Educational Expectations

The consistent results on the relatively stable relationship between RFP and adolescent development and the mixed results on the relationship between AFP and adolescent development both call for research on the underlying mechanism. Although a few studies have explored the mediating mechanism from adolescents’ filial piety to their academic achievement or subjective wellbeing, most of them have focused on the role of adolescents’ goals and self-schemata about capacities, such as mindset, goal orientations, autonomy, and emotional intelligence (Chen et al., 2018; Jen et al., 2019; Sun et al., 2019; Wu and Chen, 2020; Zhou et al., 2020). To the best of our knowledge, little research has focused on adolescents’ expectancies for success, which could be a more proximal determinant than goals and self-schemata for adolescent academic and psychological outcomes.

According to situated expectancy–value theory (Eccles and Wigfield, 2020), achievement-related outcomes are most directly influenced by individuals’ expectancies of success (e.g., educational expectations), that is, individuals’ beliefs about how well they will do on upcoming tasks. Moreover, this theory focuses on the socialization processes that lead to individual differences in expectancies, and it underscores the role of the cultural milieu. That is, the cultural milieu influences individuals’ expectancies by shaping their self-schemata, thereby contributing to achievement-related outcomes. Accordingly, adolescents’ filial piety may relate to academic achievement and subjective wellbeing through their own educational expectations.

Educational expectations are realistic beliefs regarding future academic outcomes, such as the highest level of educational attainment (Yamamoto and Holloway, 2010; Wang and Benner, 2014; Ren et al., 2021). Adolescents’ educational expectations are usually measured by asking them which educational level they expect to complete (OECD, 2019; Ren et al., 2021). The high educational expectations of adolescents have been demonstrated to be important factors for their academic and psychological adjustment (Abu-Hilal, 2000; Jacob and Wilder, 2011; Phillips, 2012; Chai et al., 2020). For example, the results of the Programme for International Students Assessment (PISA) 2015 showed that 15-year-old students’ expectation to complete a university education was strongly related to their life satisfaction; moreover, top-performing students were found to be 3.5 times more likely to hold the expectation that they would earn a university degree than were low performers (OECD, 2017). A similar trend was found in Beijing, Shanghai, Jiangsu, and Guangdong, China, which participated in the PISA 2015 (OECD, 2017). Several studies involving Chinese adolescents have also reported the positive effects of adolescents’ educational expectations on their academic achievement and subjective wellbeing (Long and Pang, 2016; OECD, 2017; Guo et al., 2018; Lv et al., 2018; Chai et al., 2020; Wu et al., 2020; Ren et al., 2021).

Cross-cultural studies have shown that Asian students, including Chinese students, tend to hold higher expectations for future education than do their North American and European counterparts (Hao and Bonstead-Bruns, 1998; Goyette and Xie, 1999; OECD, 2019). Moreover, the relatively higher educational expectations of Asian students are believed to be related to Confucian values in Asian societies (Hong and Salili, 2000; Li and Xie, 2020). In addition to the emphasis on educational effort and attainment, the emphasis on intergenerational interdependence in Confucian values has been regarded as having an important function in promoting children’s educational expectations (Goyette and Xie, 1999; Hsin and Xie, 2014; Tao, 2016; Li and Xie, 2020). That is, just as parents should raise and educate their children, children have the obligation to repay their parents and care for them as they age. Academic success is regarded as an essential way of honoring family (Kim and Park, 2006; Pomerantz et al., 2011), and the financial benefits from a tertiary education can provide better material support for the family. Accordingly, adolescents with higher filial piety may have stronger motivations to attain higher educational levels. Previous studies have shown positive relationships of filial piety with academic motivation and achievement goal orientations among Chinese adolescents (Chow and Chu, 2007; Hui et al., 2011; Chen, 2016). However, since students with AFP are academically motivated by external incentives or pressure (avoiding punishment and obeying parents’ demands; Bedford and Yeh, 2019; Ryan and Deci, 2020), they may hold negative academic self-attitudes, although they appear to make an effort in school learning (Goodman et al., 2011; Li, 2014). Accordingly, AFP may also have a negative effect on adolescents’ educational expectations.

### Moderating Effect of Parents’ Educational Expectations

From the perspective of situated expectancy–value theory, parents play a crucial role in the processes of children’s socialization of expectancies (Eccles and Wigfield, 2020). Children usually take their parents’ educational expectations for them as an important reference for their own expectations for future education (Wu et al., 2018; Guo et al., 2021b). Moreover, parents’ high educational expectations are highly associated with parents’ emphasis on the importance of education and deep involvement in children’s academic activities (Yamamoto and Holloway, 2010), which function as an “environmental cue” and indirectly boost children’s emphasis on academic achievement (Wu et al., 2018). Thus, when parents hold high educational expectations for their children, children are more likely to take academic success as a way of honoring and returning respect to their parents, and, under this circumstance, children with high filial piety may hold higher levels of educational expectations. Some indirect evidence shows that parents’ educational expectations and positive parent–adolescent relationships interact to affect adolescents’ educational expectations (Wu et al., 2018); that is, adolescents who perceive warm relationships with parents have higher educational expectations, especially when their parents hold high educational expectations. Therefore, a high level of parents’ educational expectations could enhance the effect of children’s filial piety on their educational expectations.

Parents’ educational expectations may also buffer the relationship between adolescents’ educational expectations and developmental outcomes. According to person–environment fit theory (Edwards et al., 1998; Sagiv and Schwartz, 2000; Etzel and Nagy, 2016), an individual’s motivation, behavior, and wellbeing are influenced by the fit between the characteristics of the individual and the environment, with the optimal adjustment occurring when there is a match. Parents’ high educational expectations reflect the fact that parents value learning and achievement, have positive attitudes toward their children’s ability, and provide more academic support for their children (Yamamoto and Holloway, 2010; Long and Pang, 2016; Li et al., 2019). In such family environments, the academic and psychological demands of adolescents with high self-expectations can be satisfied; therefore, the positive effects of adolescents’ educational expectations on academic and psychological outcomes can be enhanced. For example, Hao and Bonstead-Bruns (1998) found that shared expectations between parents and adolescents had a positive impact on adolescents’ academic achievement, indicating that adolescents were more likely to earn high grades when both the adolescents and their parents held high expectations for the adolescents’ future education. Similarly, Almroth et al. (2018) found that the odds of adolescents showing externalizing behaviors decreased dramatically when both the adolescents and their parents expected a university education compared with either the adolescents or the parents having expectations that were lower than a university education. Although these two studies were not conducted with Chinese adolescents, the positive role of parents’ educational expectations in adolescents’ academic and psychological outcomes that existed in these studies has also been found in Chinese society (Long and Pang, 2016; Guo et al., 2018; Lv et al., 2018; Leung and Shek, 2019; Lu et al., 2020; Ren et al., 2021). Moreover, the suitability has been demonstrated of person–environment fit theory for Chinese society (Jiang and Jiang, 2015; Wang and Wang, 2018; Liu et al., 2019).

### Current Study

**Figure 1:**
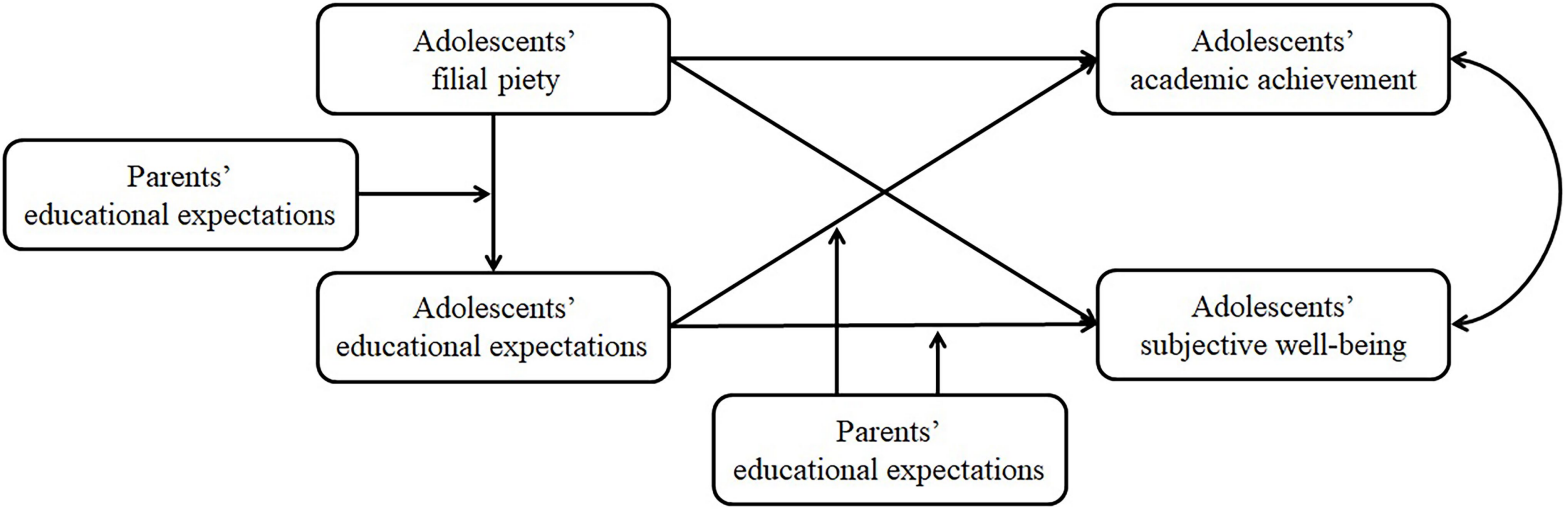
The hypothesized moderated mediation model.

In summary, although a few studies have explored the relationship of filial piety to the academic achievement or life satisfaction of Chinese adolescents, given the positive relationship between academic achievement and subjective wellbeing (Bücker et al., 2018; Wu et al., 2020), it is necessary to examine whether filial piety still has an effect on academic achievement and on subjective wellbeing while controlling for its effect on the other outcome factor. Moreover, the possible moderated mediation effect of educational expectations on these relationships needs to be examined. The purpose of this study is to clarify the effects of adolescents’ filial piety on their academic achievement and subjective wellbeing by considering the two outcomes simultaneously, to examine the mediating role of adolescents’ educational expectations in the above relationships, and to then explore the moderating role of parents’ educational expectations in the relationships between adolescents’ filial piety and educational expectations, and between adolescents’ educational expectations and their academic achievement and subjective wellbeing. By addressing these issues, this study may further verify the significance of filial piety for adolescent development and contribute to a better understanding of how and when filial piety influences adolescent development. The hypothesized model is presented in Figure 1. Specifically, this study hypothesizes the following:

RFP is positively related to academic achievement (H1a) and subjective wellbeing (H2a) when the two outcomes are considered simultaneously.
AFP is negatively related to academic achievement (H1b) but positively related to subjective wellbeing (H2b) when the two outcomes are considered simultaneously.
Adolescents’ educational expectations mediate the relationship between RFP and both academic achievement (H3a) and subjective wellbeing (H4a), and they mediate the relationship between AFP and both academic achievement (H3b) and subjective wellbeing (H4b).
Parents’ educational expectations positively moderate the effect of RFP on adolescents’ educational expectations (H5a) and of AFP on adolescents’ educational expectations (H5b).
Parents’ educational expectations positively moderate the effect of adolescents’ educational expectations on their academic achievement (H6a) and subjective wellbeing (H6b).

## Materials and Methods

### Participants and Procedure

The participants were 677 sixth graders and their parents, who were recruited from five primary schools in Beijing, China. School approval and informed consent from the parents were obtained prior to the survey. The students completed standardized academic achievement tests in reading and mathematics and a questionnaire administered in their regular classrooms by two trained research assistants. The academic achievement tests and the questionnaire were administered in three separate 45-min sessions. The mothers and fathers completed questionnaires individually at home. A total of 26 students had missing responses on academic achievement or educational expectations, and of the remaining students, 49 mothers and 75 fathers did not return the questionnaires. Thus, the final sample included 651 students and their parents (603 mothers and 577 fathers). Of the 651 students, 348 (53.5%) were boys, and 303 (46.5%) were girls; the mean age was 12.24 years (*SD* = 0.36). The mean ages of the mothers and fathers were 39.66 years (*SD* = 3.99) and 41.98 years (*SD* = 4.66), respectively. Of the 603 mothers, 19.9% had not finished high school, 53.8% had a high school diploma, 22.4% had a 4-year college degree, and 3.9% had an education beyond the 4-year college level. Of the 577 fathers, 18.2% had not finished high school, 47.6% had a high school diploma, 23.7% had a 4-year college degree, and 10.5% had an education beyond the 4-year college level. This study was approved by the Institutional Review Board of the Collaborative Innovation Center of Assessment toward Basic Education Quality, Beijing Normal University. Given that the measures used in this study have been shown to have acceptable reliability and validity in Chinese adolescent samples, no pilot study was undertaken.

### Measures

#### Filial Piety

Students’ filial piety was measured by the Dual Filial Piety Scale-Chinese version (DFPS; Yeh and Bedford, 2003). The original scale includes 16 items, and each item is answered on a 6-point scale ranging from 1 (*strongly disagree*) to 6 (*strongly agree*). For this study, the scale was modified for use with Chinese mainland students by removing two items (“Hurry home upon the death of a parent, regardless of how far away you live” and “To continue the family line, one must have at least a son”). Of the remaining 14 items, 7 measured RFP, and 7 measured AFP. In this study, Cronbach’s *α* was 0.85 and 0.84 for RFP and AFP, respectively.

#### Academic Achievement

Academic achievement was measured using standardized reading and math achievement tests, which were developed by the National Children’s Study of China (NCSC) project (Dong and Lin, 2011). For reading achievement, there were 33 multiple-choice items, and for math achievement, there were 26 multiple-choice items and 6 constructed-response items. Higher scores represented higher academic achievement.

#### Subjective Wellbeing

Subjective wellbeing was conceptualized as life satisfaction, positive affect, and negative affect. Life satisfaction was measured by a single item (Overall, how satisfied are you with your present life?) answered on a 7-point scale ranging from 1 (*strongly dissatisfied*) to 7 (*strongly satisfied*; Huebner et al., 2006). Positive affect and negative affect were measured by the Chinese Revision of the Positive and Negative Affect Scale (Qiu et al., 2008) originally developed by Watson et al. (1988). The revised scale included 18 descriptive terms for positive affect and negative affect experience (such as excited and afraid). The participants were asked to report the affect they experienced over the last 2 weeks on a 5-point scale ranging from 1 (*very slightly or not at all*) to 5 (*extremely*). In this study, Cronbach’s *α* was 0.89 and 0.90 for positive affect and negative affect, respectively.

#### Educational Expectations

The students reported their educational expectations (“As things stand now, how far in school do you think you will get?”) on a 6-point scale from 1 (*primary school or below*) to 6 (*master’s degree or above*). The mothers and fathers reported their educational expectations for their children (“As things stand now, how far in school do you think your child will get?”) using the same response categories.

#### Demographics

Demographic information was obtained from the mothers or fathers and included the student’s gender, family income, and the mother’s and father’s educational levels.

### Analysis Plan

First, a descriptive statistical analysis and a Pearson bivariate correlation analysis among all variables were conducted by IBM SPSS Statistics 19.0. Second, structural equation modeling (SEM) was performed by Mplus 7.11, which was used to examine the relationships between adolescents’ filial piety and their academic achievement and subjective wellbeing, the mediating effect of adolescents’ educational expectations on these relationships, and the hypothesized moderated mediation model. All continuous variables were standardized before being entered into the models for statistical comparability and the interpretability of scores. Academic achievement was treated as a latent variable, with the reading and math test scores serving as indicators. Subjective wellbeing was also treated as a latent variable, with life satisfaction, positive affect, and negative affect serving as indicators. All other variables were treated as manifest variables. RFP and AFP, and academic achievement and subjective wellbeing were allowed to be correlated in the model. When the mediating role was tested of adolescents’ educational expectations, bias-corrected bootstrapping with 5,000 samples and 95% confidence intervals (Cis) was applied to examine the significance of the mediating effect. Moreover, the effect size for the indirect effect was calculated by the ratio of the indirect effect to the total effect (Wen and Fan, 2015). When testing the moderating effect of parents’ educational expectations, adolescents’ filial piety and parents’ (fathers or mothers’) educational expectations were multiplied to create an interaction term for predicting adolescents’ educational expectations, and adolescents’ and parents’ (fathers or mothers’) educational expectations were multiplied to create an interaction term for predicting academic achievement and subjective wellbeing. The simple slope test was used to interpret the interaction effect. Adolescents’ gender and family income and parents’ educational levels were controlled in all models. Missing data were handled by using full information maximum likelihood (FIML) estimation. According to Hu and Bentler (1999), the model is acceptable when the confirmatory fit index (CFI) > 0.90, Tucker–Lewis index (TLI) > 0.90, and root mean square error of approximation (RMSEA) < 0.08.

## Results

### Preliminary Analysis

The descriptive statistics and zero-order correlations among all study variables are presented in Table 1. Adolescents’ RFP was positively associated with their and their parents’ reports of educational expectations, academic achievement, life satisfaction, and positive affect, and it was negatively associated with negative affect. Furthermore, adolescents’ AFP was negatively associated with parents’ educational expectations and academic achievement and positively associated with negative affect.

**Table 1:** Descriptive statistics and intercorrelations for all study variables.

	1	2	3	4	5	6	7	8	9	10	11	12	13
1. Reciprocity filial piety	–												
2. Authoritarian filial piety	0.42^**^	–											
3. Adolescent’s educational expectations	0.17^**^	−0.02	–										
4. Mother’s educational expectations	0.14^**^	−0.09^*^	0.44^**^	–									
5. Father’s educational expectations	0.14^**^	−0.10^*^	0.38^**^	0.74^**^	–								
6. Reading achievement	0.09^*^	−0.28^***^	0.36^***^	0.40^***^	0.38^***^	–							
7. Math achievement	0.10^**^	−0.16^***^	0.38^***^	0.40^***^	0.40^***^	0.60^***^	–						
8. Life satisfaction	0.41^**^	0.20^**^	0.16^**^	0.06	0.09^*^	−0.03	0.03	–					
9. Positive affect	0.38^**^	0.20^**^	0.27^**^	0.12^**^	0.13^**^	0.04	0.12^**^	0.48^**^	–				
10. Negative affect	−0.17^**^	0.00	−0.19^**^	−0.10^*^	−0.10^*^	−0.19^***^	−0.22^***^	−0.38^**^	−0.32^**^	–			
11. Gender	−0.02	−0.15^**^	0.03	0.04	0.04	0.13^**^	−0.03	−0.12^**^	−0.09^*^	0.02	–		
12. Income	0.03	−0.06	0.21^**^	0.31^**^	0.29^**^	0.18^***^	0.20^***^	0.04	0.04	−0.03	−0.01	–	
13. Mother’s education	0.01	−0.08	0.29^**^	0.34^**^	0.30^**^	0.20^***^	0.18^***^	0.05	0.09^*^	−0.01	0.02	0.40^**^	–
14. Father’s education	0.02	−0.10^*^	0.32^**^	0.39^**^	0.36^**^	0.26^***^	0.23^***^	−0.01	0.03	−0.03	0.03	0.45^**^	0.72^**^

^*^p < 0.05, ^**^p < 0.01, ^***^p < 0.001.

### Direct Effects

A direct effect model was tested with only direct paths from RFP and AFP to academic achievement and subjective wellbeing. The model showed an excellent fit to the data [*χ*^2^ (29) = 97.39, CFI = 0.95, TLI = 0.93, RMSEA = 0.04]. Adolescents’ RFP and AFP were positively correlated with each other (*r* = 0.41, *p* < 0.001), while their academic achievement and subjective wellbeing were not significantly correlated with each other (*r* = −0.04, *p* < 0.05). Adolescents’ RFP positively predicted academic achievement (*β* = 0.29, *p* < 0.001) and subjective wellbeing (*β* = 0.54, *p* < 0.001). In addition, adolescents’ AFP negatively predicted academic achievement (*β* = −0.37, *p* < 0.001), but did not significantly predict subjective wellbeing (*β* = 0.02, *p* > 0.05).

### Mediating Effects

**Figure 2:**
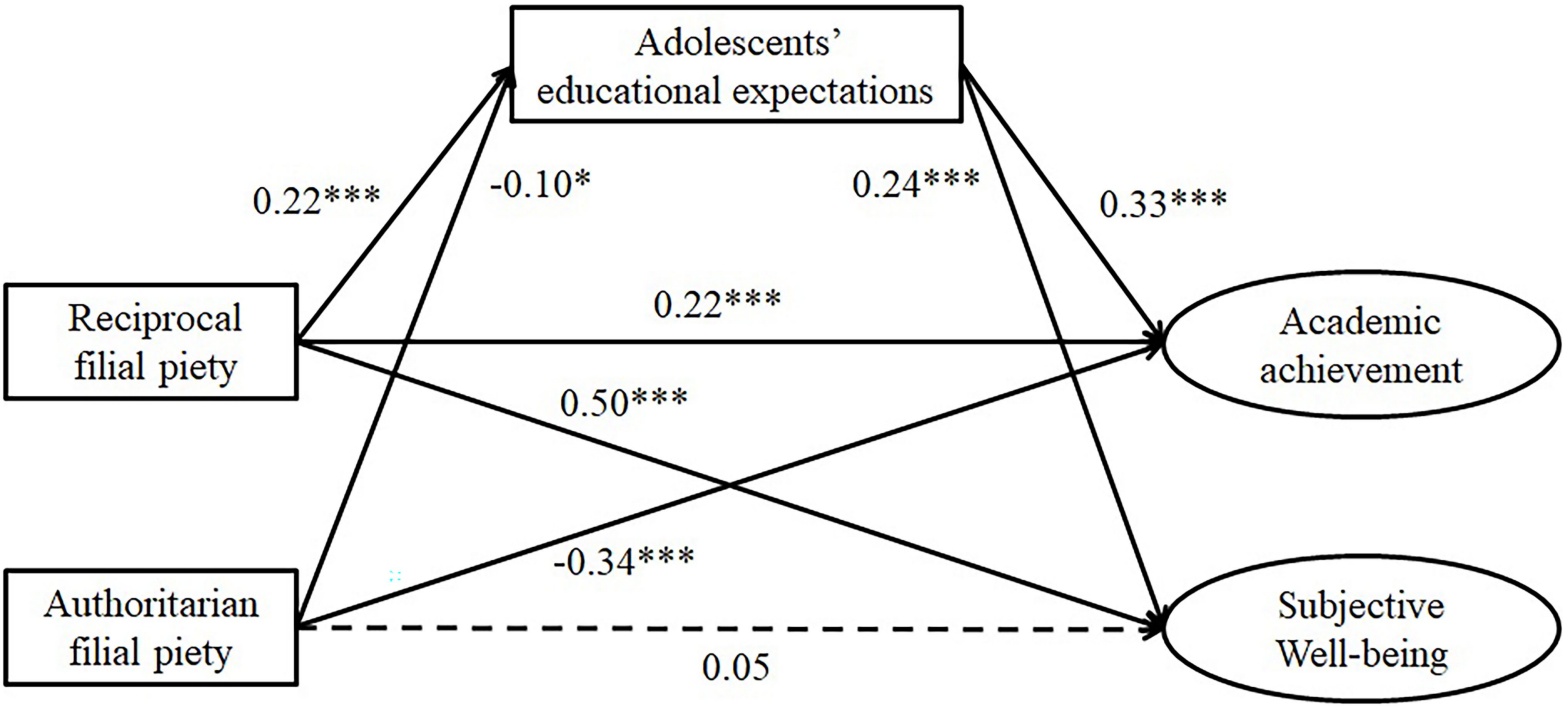
Standardized coefficients for the mediating role of adolescents’ educational expectations in the relationships between filial piety and academic achievement and subjective wellbeing.

A mediation model was tested with adolescents’ educational expectations as a mediator in the relationships between filial piety and academic achievement and subjective wellbeing (see Figure 2). The model showed an excellent fit to the data [*χ*^2^ (32) = 117.41, CFI = 0.94, TLI = 0.91, RMSEA = 0.04]. As shown in Figure 2, adolescents’ RFP positively predicted their educational expectations, while their AFP negatively predicted their educational expectations. In turn, adolescents’ educational expectations positively predicted their academic achievement and subjective wellbeing.

The results of the bootstrapping analyses and the effect size for the indirect and direct effects are presented in Table 2. A 95% CI that did not include zero indicated significant mediation. Accordingly, all four indirect effects *via* adolescents’ educational expectations were significant, although the effect size was not very large.

**Table 2:** Testing mediation using bootstrapping analyses.

Indirect paths	Indirect effect	95%CIs	Effect size
Reciprocal filial piety → Adolescents’ educational expectations → Academic achievement	0.057^***^	0.034 ~ 0.089	0.249
Reciprocal filial piety → Adolescents’ educational expectations → Subjective wellbeing	0.039^**^	0.021 ~ 0.069	0.096
Authoritarian filial piety → Adolescents’ educational expectations → Academic achievement	−0.027^*^	−0.051 ~ −0.007	0.089
Authoritarian filial piety → Adolescents’ educational expectations → Subjective wellbeing	−0.018^*^	−0.037 ~ −0.006	—^a^

Results presented the unstandardized coefficient. ^*^p < 0.05, ^**^p < 0.01, ^***^p < 0.001.

a *The effect size for the indirect effect from authoritarian filial piety to subjective wellbeing was inapplicable since the indirect effect (−) and the direct effect (+) have opposite signs (Wen and Fan, 2015)*.

### Moderated Mediation Effects

The moderating role of parents’ educational expectations in the relationship between adolescents’ filial piety and their educational expectations was examined first. Moreover, fathers’ and mothers’ educational expectations were tested separately. For the moderating role of fathers’ educational expectations, the model showed a good fit to the data [*χ*^2^ (39) = 146.90, CFI = 0.91, TLI = 0.90, RMSEA = 0.07]. Fathers’ educational expectations positively predicted adolescents’ educational expectations (*β* = 0.27, *p* < 0.001), but neither the interaction of fathers’ educational expectations and adolescents’ RFP (*β* = 0.01, *p* > 0.05) nor the interaction of fathers’ educational expectations and adolescents’ AFP (*β* = 0.04, *p* > 0.05) were significant.

For the moderating role of mothers’ educational expectations, the model showed a good fit to the data [*χ*^2^ (39) = 158.32, CFI = 0.90, TLI = 0.89, RMSEA = 0.07]. Mothers’ educational expectations positively predicted adolescents’ educational expectations (*β* = 0.34, *p* < 0.001), but neither the interaction of mothers’ educational expectations and adolescents’ RFP (*β* = 0.07, *p* > 0.05) nor the interaction of mothers’ educational expectations and adolescents’ AFP (*β* = −0.04, *p* > 0.05) were significant.

Then, the moderating role of parents’ educational expectations in the relationship between adolescents’ educational expectations and their academic achievement and subjective wellbeing was examined. For the moderating role of fathers’ educational expectations, the model showed a good fit to the data [*χ*^2^ (44) = 180.64, CFI = 0.91, TLI = 0.90, RMSEA = 0.06]. Fathers’ educational expectations positively predicted adolescents’ academic achievement (*β* = 0.30, *p* < 0.001) but did not significantly predict subjective wellbeing (*β* = 0.04, *p* > 0.05). In addition, the interaction of fathers’ and adolescents’ educational expectations did not significantly predict academic achievement (*β* = −0.04, *p* > 0.05) or subjective wellbeing (*β* = 0.08, *p* = 0.09).

**Figure 3:**
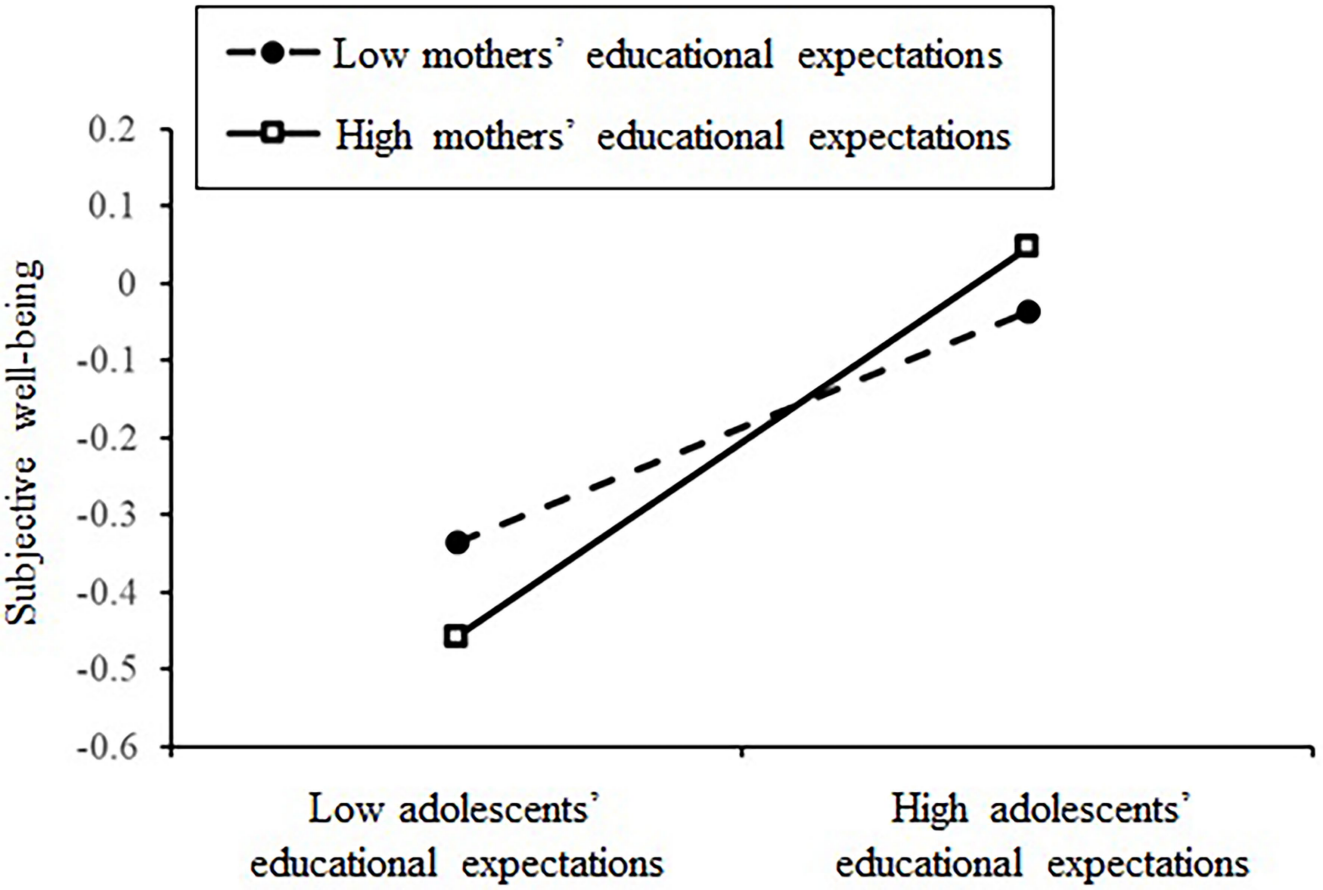
Moderating role of mothers’ educational expectations in the relationship between adolescents’ educational expectations and subjective wellbeing.

For the moderating role of mothers’ educational expectations, the model showed a good fit to the data [*χ*^2^ (44) = 204.78, CFI = 0.91, TLI = 0.90, RMSEA = 0.06]. Mothers’ educational expectations positively predicted adolescents’ academic achievement (*β* = 0.32, *p* < 0.001) but did not significantly predict subjective wellbeing (*β* = −0.01, *p* > 0.05). In addition, the interaction of mothers’ and adolescents’ educational expectations did not significantly predict academic achievement (*β* = −0.07, *p* > 0.05) but positively predicted subjective wellbeing (*β* = 0.09, *p* < 0.05). The results of a simple slope test (see Figure 3) showed that when mothers’ educational expectations were high (+1 *SD*), adolescents’ educational expectations positively predicted their subjective wellbeing and were stronger (*simple slope* = 0.252, *SE* = 0.046, *p* < 0.001), and when mothers’ educational expectations were low (−1 *SD*), adolescents’ educational expectations positively predicted their subjective wellbeing but were weaker (*simple slope* = 0.151, *SE* = 0.040, *p* < 0.001).

Furthermore, the moderating role of mothers’ educational expectations on the indirect effect between filial piety and subjective wellbeing *via* adolescents’ educational expectations was examined. The results of the bootstrapping analyses are presented in Table 3. The indirect effect of RFP on subjective wellbeing was significant both at high (+1 *SD*) and low (−1 *SD*) levels of mothers’ educational expectations, but the effect was weaker at the low than at high levels. Similarly, the indirect effect of AFP on subjective wellbeing was significant at both the high and low levels of mothers’ educational expectations, but the effect was weaker at the low than it was at the high levels.

**Table 3:** Testing moderated mediation using bootstrapping analyses.

Indirect paths	Indirect effect	95%CIs
High mothers’ educational expectations (+1 *SD*)		
Reciprocal filial piety → Adolescents’ educational expectations → Subjective wellbeing	0.056^**^	0.027 ~ 0.099
Authoritarian filial piety → Adolescents’ educational expectations → Subjective wellbeing	−0.023^*^	−0.049 ~ −0.006
Low mothers’ educational expectations (−1 *SD*)		
Reciprocal filial piety → Adolescents’ educational expectations → Subjective wellbeing	0.034^*^	0.016 ~ 0.060
Authoritarian filial piety → Adolescents’ educational expectations → Subjective wellbeing	−0.014^*^	−0.030 ~ −0.003

Results presented the unstandardized coefficient. ^*^p < 0.05, ^**^p < 0.01.

## Discussion

This study investigated how Chinese early adolescents’ filial piety is related to their academic achievement and subjective wellbeing, and it examined the mediating role of adolescents’ educational expectations in these relationships and the moderating role of parents’ educational expectations in the relationship of adolescents’ educational expectations to academic achievement and subjective wellbeing. The findings support the associations of filial piety with academic achievement and subjective wellbeing. In addition, adolescents’ educational expectations play a mediating role in the relationship between filial piety and adolescents’ development. Furthermore, the study found that parental educational expectations moderate the relationship between adolescents’ educational expectations and their subjective wellbeing.

The first main finding is the effects of filial piety on adolescents’ academic achievement and subjective wellbeing. As expected, when the two outcomes were considered simultaneously, RFP was positively related to adolescents’ academic achievement and subjective wellbeing, supporting H1a and H2a and consistent with previous findings (Chen and Wong, 2014; Chen, 2016; Sun et al., 2019; Leung and Shek, 2020; Zhou et al., 2020). The spontaneous love and gratitude for parents implied in RFP promote students’ initiative and autonomous learning, which contribute to better academic achievement (Chen and Wong, 2014; Chen, 2016). Meanwhile, adolescents with RFP usually have a positive relationship with their parents that can satisfy their needs for interpersonal relatedness (Tay and Diener, 2011; Bedford and Yeh, 2019, 2021), which leads to a higher level of happiness in adolescents. The results on the positive effect of RFP on academic and psychological outcomes in Chinese culture are also in line with research on parent–child interactions in other cultural settings (Steinberg and Silk, 2002; Rutten et al., 2016), which demonstrates the importance of affection and emotional support for adolescent development.

Moreover, when the two outcomes were both considered, AFP was found to be negatively related to academic achievement, supporting H1b and in line with previous findings (Chen and Wong, 2014; Chen, 2016). The findings indicated that the negative effect of AFP on academic success was more prominent than the positive effect in the context of modern Chinese society. Bringing honor to the family is still recognized as an important attribute of filial piety in contemporary society (Yeh et al., 2013), while adolescents’ motivation aroused by it is passive and extrinsic, which does not have a long-lasting positive effect on academic achievement (Legault et al., 2006; Chen and Ho, 2012). Moreover, due to the social and economic changes in China, the importance is decreasing of passive submission and obedience to parents, and the emphasis is strengthening on adolescent autonomous development (Bush et al., 2002; Yeh, 2007). Thus, the self-suppression and self-sacrifice embedded in AFP are not adapted to current conditions; thereby, its harmful effects are particularly significant. Our findings indicate that the authoritarian elements of filial piety have certain negative implications for adolescent academic development from a contemporary cultural perspective.

However, AFP was found to be not significantly related to adolescents’ subjective wellbeing; thus, H2b is not supported. The different dimensions of AFP may very likely have different effects on adolescent psychological development (Guo et al., 2021a). On the one hand, AFP is associated with responsibility and could satisfy the need for a collective identity toward society (Bedford and Yeh, 2021). Especially for adolescents who are in an important stage of identity formation, the endorsement of obedience norms could enable them to gain positive feedback from others, such as other family members, neighbors, and teachers. Therefore, AFP has a positive effect on adolescent psychological development. For example, the correlation results in the present study showed positive associations of AFP with life satisfaction and positive affect. However, given that the status is unequal between parents and children who obey without question, AFP hinders the development of individuating autonomy (Pan et al., 2013; Yeh, 2014) and thus has a negative effect on adolescent psychological development, such as low self-esteem and depressed mood (Yeh, 2006; Bynum and Kotchick, 2006; Rutten et al., 2016). Accordingly, the expected positive effect of AFP on subjective wellbeing may be offset by its negative effect. This factor might also explain the mixed findings on AFP and life satisfaction. That is, in some cases, the beneficial aspect of AFP for life satisfaction is dominant, while in other cases, the harmful aspect becomes more apparent. Future research should aim to detect individual or environmental variables that can buffer the relationship between AFP and life satisfaction or other forms of subjective wellbeing. Our findings indicate that although horizontal filial relations are gradually unaccepted, AFP may still have positive implications for adolescent wellbeing to a certain degree.

The second main finding is that the relationships of AFP and RFP to academic achievement and subjective wellbeing were all mediated by adolescents’ educational expectations, supporting H3a, H4a, H3b, and H4b. The results demonstrate that educational expectations play an important role in the process in which individuals’ self-schemata influence their achievement-related outcomes, supporting situated expectancy–value theory (Eccles and Wigfield, 2020). The results show that RFP is positively associated with adolescents’ educational expectations. RFP emphasizes psychological fulfillment in many ways, such as autonomy and relatedness. All of these factors promote adolescents’ competence and self-worth, which are critical for the development of adolescents’ self-esteem (Yan and Chen, 2018). For example, a previous study found that RFP is positively related to adolescents’ self-esteem (Jen et al., 2019). Higher self-esteem predicts positive expectations about adolescents’ future (Kim and Kim, 2013). In addition, adolescents with strong RFP have emotional safety and affective bonding with their parents (Bedford and Yeh, 2021); hence, they tend to repay their parents with genuine affection and thus have higher expectations about their future academic achievements, which are regarded as a primary way of repaying parents (Ho, 1986; Fwu et al., 2016). All these factors shape the strong relationship between RFP and educational expectations, which in turn contributes to better academic achievement and subjective wellbeing.

However, the situation is different for AFP. AFP is negatively related to adolescents’ educational expectations. One possible reason is that honoring the family is an external motivator for adolescents to pursue academic success, and students with external regulation motivation are more likely to have external loci of control and to lack autonomy (Guay et al., 2000; Ryan and Deci, 2020). Both of these factors have been found to be related to low expectations of success (Coleman and DeLeire, 2003; Kenny et al., 2010). Moreover, AFP emphasizes prioritizing others’ wishes over personal wishes. Continuous self-suppression and self-sacrifice result in negative self-cognition, such as low self-esteem and self-efficacy (Yeh, 2006; Wong et al., 2010), and negative self-cognition leads to negative expectations about the future (Patton et al., 2004; Ma et al., 2018). This result further provides a mechanistic explanation for the negative relationship between AFP and academic performance. Although previous studies provide some evidence that AFP may contribute to student academic motivation and performance-based goals (Chow and Chu, 2007; Chen, 2016), the present study found that students with AFP do not set high educational goals for themselves, nor do they have strong expectations for success. Accordingly, AFP showed a negative relationship with adolescent academic achievement.

Parental educational expectations did not enhance the relationship between filial piety and adolescents’ educational expectations, not supporting H5a and H5b. To the best of our knowledge, this is the first study to explore the moderating effect of parental expectations on the above relationships. One possible reason is that education is highly valued in China because it is considered to contribute to upward mobility, better jobs and incomes, and even better marriage prospects (Wu and Treiman, 2007; Huang and Gove, 2015). Accordingly, Chinese parents attach importance to education and generally have high educational expectations (Li, 2001; Zou et al., 2013; Guo, 2014). Therefore, the variation in parents’ educational expectations may be too small to trigger the moderating effect. Similarly, another possible reason is that, due to the emphasis on education, Chinese adolescents also have high expectations for their own future education (Archer and Francis, 2006). Although parental educational expectations could enhance the association between filial piety and adolescents’ educational expectations to some degree, there may be a ceiling effect on adolescents’ educational expectations, masking the moderating effect that would otherwise have existed. In addition, it is likely that parents’ educational expectations affect children’s expectations more directly other than *via* a moderating mechanism. Previous research has revealed a strong intergenerational transmission of educational expectations, even after controlling for several potential indirect mechanisms (Wu et al., 2018; Guo et al., 2021b). This unexpected finding indicated that the moderating effect of parental educational expectations on the relationship between filial piety and adolescents’ educational expectations seems more complicated than expected and requires further investigation.

However, mothers’ educational expectations could moderate the relationship between adolescents’ educational expectations and their subjective wellbeing, although mothers’ educational expectations were not directly related to adolescents’ subjective wellbeing. Specifically, the positive relationship between adolescents’ educational expectations and subjective wellbeing was stronger under a higher level of mothers’ educational expectations, supporting H6b, and this result is consistent with person–environment fit theory (Sagiv and Schwartz, 2000). Fathers’ educational expectations also showed a marginally significant moderating effect on the relationship between adolescents’ educational expectations and their subjective wellbeing. The weaker effect is probably due to the lower involvement of fathers in the care of children (Bianchi and Milkie, 2010). High parental expectations indicate that parents value their children’s academic achievement and have positive evaluations of their children’s capacity (Yamamoto and Holloway, 2010). Thus, adolescents receiving higher parental educational expectations may obtain more approval and support and even have higher self-efficacy, which may contribute to better mental health. This suggestion is consistent with previous findings (Almroth et al., 2018). Research on person–organization value fit also found that parent–child value congruence was associated with children’s better wellbeing (Flurry et al., 2021).

In addition, although this study found a significant relationship between parental educational expectations and academic achievement, which is consistent with previous findings (Pinquart and Ebeling, 2020), there was no significant moderating effect of parental educational expectations on the relationship between adolescents’ educational expectations and academic achievement; thus, H6a was not supported. One possible explanation may be that when puberty is reached, adolescents become more independent and distance themselves from their parents (Daniels, 1990). Family influences begin to decline, and school factors, such as peer groups, become more important. Therefore, adolescents are less likely to depend on parental involvement to enhance their learning motivation or academic achievement, and sometimes, they may even hope that their parents will become less involved or resist their support (Coleman and McNeese, 2009). Accordingly, adolescents’ expectations or goals increasingly rely on their own perceptions of their current status (Aceves et al., 2020), and the relationship between their educational expectations and academic achievement is less likely to be affected by their parents’ educational expectations. This result is similar to the findings of Liu et al. (2013), who found that the relationship between autonomous motivation and creative thinking cannot be strengthened by high paternal involvement in education.

The major contributions of the current study are as follows. First, it adds to the existing literature on the effects of filial piety on adolescents’ development. In particular, the current study simultaneously considers academic achievement and subjective wellbeing, which could clarify the unique effect of filial piety on adolescents’ academic achievement and subjective wellbeing. Second, this study provides insights into how filial piety is related to adolescents’ development. To the best of our knowledge, this study is the first to focus on the mediating role of adolescents’ expectancies for success and to consider the moderating role of fathers’ and mothers’ educational expectations. In doing so, it advances research on the mechanism of filial piety. Third, previous research regarding the role of filial piety in adolescents’ development has mainly focused on late adolescents. The current study, which focuses on early adolescents, broadens the applicability of the filial piety theory and research.

These contributions suggest some practical implications for parents, schools, and education policymakers. Our findings suggest that RFP can help foster successful students by enhancing their expectancies for success, while AFP may hinder adolescent development by frustrating their expectations. Thus, parents and schools should attach importance to cultivating adolescents’ positive beliefs in filial piety and to creating an equal and close parent–child relationship. Parents and schools should also weaken adolescents’ negative beliefs, such as obedience to authority, which may have a disruptive effect on their academic achievement. Moreover, in a broader context, education policymakers should realize that, in addition to promoting policies that are directly related to disciplinary education, it is very important to pay attention to and strengthen moral education. Providing education on filial piety that emphasizes equal status and intimate relationships with parents is conducive to improving adolescents’ positive self-cognition and to forming positive development goals to promote the comprehensive development of adolescents. In addition, our findings suggest that both parents and children should establish realistic but positive beliefs about future education. Adolescents’ high educational expectations are a kind of positive self-cognition and a learning motivation, and high parental expectations are regarded as a kind of approval and support for children.

The present study is not free of limitations. First, the causal relationships among filial piety, adolescents’ educational expectations, academic achievement, and subjective wellbeing could not be determined in this cross-sectional correlational study. Longitudinal studies with more extensive follow-up are required. Second, when the mediating effect of adolescents’ educational expectations was considered, the relationships between filial piety and academic achievement and subjective wellbeing still existed. This result implies that other factors may explain how filial piety affects students’ academic achievement and subjective wellbeing, such as students’ academic self-concept and motivations and parent–adolescent relationships. Finally, the participants in this study were recruited only from Mainland China. Future studies might be needed to replicate our findings in samples from other Asian and even Western countries and regions.

In conclusion, the present study systematically investigated the relationships among filial piety, adolescents’ educational expectations, parental educational expectations, academic achievement, and subjective wellbeing among Chinese early adolescents. The results suggest that two types of filial piety, RFP and AFP, have different relationships with academic achievement and subjective wellbeing and that these effects could be partially mediated by adolescents’ educational expectations. Moreover, parental educational expectations could moderate the relationship between adolescents’ educational expectations and subjective wellbeing. Our findings highlight the importance of RFP and adolescents’ and parents’ educational expectations for academic success and wellbeing. Taken together, the findings of this study broaden our understanding of filial piety and its implications for Chinese adolescents’ academic achievement and wellbeing, and they provide the basis for valuable recommendations for practice.

## Data Availability Statement

The raw data supporting the conclusions of this article will be made available by the authors, without undue reservation.

## Ethics Statement

The studies involving human participants were reviewed and approved by the Institutional Review Board of the Collaborative Innovation Center of Assessment toward Basic Education Quality, Beijing Normal University. Written informed consent to participate in this study was provided by the participants’ legal guardian/next of kin.

## Author Contributions

Conception and design of the work was done by XG and LL. Data collection and analysis of data done by all authors. XG and JL wrote the original draft of the manuscript. XG, JL, and YN participated in the work of revision and finalization of the manuscript in the process of reviewing. LL edited and co-wrote the manuscript. All authors contributed to the article and approved the submitted version.

## Funding

This work was supported by the Major Project of National Social Science Fund of China (16ZDA229) and the Youth Project of National Social Science Fund of China (17CSH024).

## Conflict of Interest

The authors declare that the research was conducted in the absence of any commercial or financial relationships that could be construed as a potential conflict of interest.

## Publisher’s Note

All claims expressed in this article are solely those of the authors and do not necessarily represent those of their affiliated organizations, or those of the publisher, the editors and the reviewers. Any product that may be evaluated in this article, or claim that may be made by its manufacturer, is not guaranteed or endorsed by the publisher.
